# The Medical Review Visiting List Perpetual

**Published:** 1892-01

**Authors:** 


THE MEDICAL REVIEW VISITING LIST PERPETUAL.—Published by J. H. Chambers & Co., St. Louis, Mo. Price 75.

It contains 24 pages of text with 37 pages of blanks usually found in such books.

In the text may be found: Artificial Respiration, Care of Galvanic Batteries, Disinfectants, Examination of Urine, Poisons and Antidotes, Tables of Doses, Doses of Medicine for Children, Table of Equivalents, Comparison of Thermo-metric Scales, Metric System of Weights and Measures, Diet Tables for Diabetics, Diagnostic Table of Euptive Fevers.

Being perpetual, price so low renders it a cheap, useful Visiting List.

